# Association of anti‐factor Xa‐guided anticoagulation with hemorrhage during ECMO support: A systematic review and meta‐analysis

**DOI:** 10.1002/clc.24273

**Published:** 2024-05-02

**Authors:** Sasa Rajsic, Robert Breitkopf, Benedikt Treml, Dragana Jadzic, Nicole Innerhofer, Christine Eckhardt, Christoph Oberleitner, Zoran Bukumiric

**Affiliations:** ^1^ Department of Anaesthesiology and Intensive Care Medicine Medical University Innsbruck Innsbruck Austria; ^2^ Anesthesia and Intensive Care Department, Pain Therapy Service Cagliari University Cagliari Italy; ^3^ Institute of Medical Statistics and Informatics, Faculty of Medicine University of Belgrade Belgrade Serbia

**Keywords:** adverse events, anticoagulation, anti‐Xa, bleeding, ECMO, hemorrhage, monitoring

## Abstract

**Background:**

The use of extracorporeal membrane oxygenation (ECMO) is associated with complex hemostatic changes. Systemic anticoagulation is initiated to prevent clotting in the ECMO system, but this comes with an increased risk of bleeding. Evidence on the use of anti‐Xa‐guided monitoring to prevent bleeding during ECMO support is limited. Therefore, we aimed to analyze the association between anti‐factor Xa‐guided anticoagulation and hemorrhage during ECMO.

**Methods:**

A systematic review and meta‐analysis was performed (up to August 2023). PROSPERO: CRD42023448888.

**Results:**

Twenty‐six studies comprising 2293 patients were included in the analysis, with six works being part of the meta‐analysis. The mean anti‐Xa values did not show a significant difference between patients with and without hemorrhage (standardized mean difference −0.05; 95% confidence interval [CI]: −0.19; 0.28, *p* = .69). We found a positive correlation between anti‐Xa levels and unfractionated heparin dose (UFH; pooled estimate of correlation coefficients 0.44; 95% CI: 0.33; 0.55, *p* < .001). The most frequent complications were any type of hemorrhage (pooled 36%) and thrombosis (33%). Nearly half of the critically ill patients did not survive to hospital discharge (47%).

**Conclusions:**

The most appropriate tool for anticoagulation monitoring in ECMO patients is uncertain. Our analysis did not reveal a significant difference in anti‐Xa levels in patients with and without hemorrhagic events. However, we found a moderate correlation between anti‐Xa and the UFH dose, supporting its utilization in monitoring UFH anticoagulation. Given the limitations of time‐guided monitoring methods, the role of anti‐Xa is promising and further research is warranted.

## Graphical abstract

There is a moderate correlation between anti‐Xa and the unfractionated heparin dose (UFH) dose, supporting its utilization in monitoring UFH anticoagulation in extracorporeal membrane oxygenation patients. Considering the contemplative limitations of time‐guided monitoring tools, the role of anti‐Xa is promising and further research including recommendations are warranted.

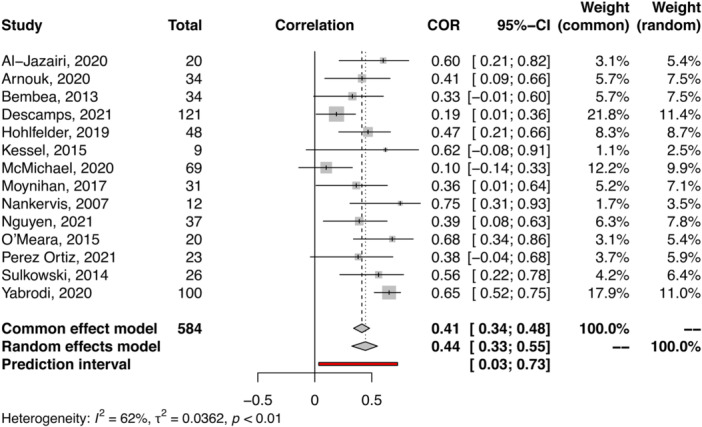

**Abbreviations**
ACT: activated clotting time
aPTT: activated partial thromboplastin time
ECMO: extracorporeal membrane oxygenation
ECPR: extracorporeal cardiopulmonary resuscitation
ELSO: Extracorporeal Life Support Organization
ICU: intensive care unit
NOS: Newcastle‐Ottawa scale score
UFH: unfractionated heparin
va‐ECMO: venoarterial extracorporeal membrane oxygenation
vv‐ECMO: venovenous extracorporeal membrane oxygenation

## 1 INTRODUCTION

The use of extracorporeal membrane oxygenation (ECMO) is associated with intricate coagulation and inflammatory changes, necessitating therapeutic anticoagulation.^1^, ^2^, ^3^, ^4^ However, systemic anticoagulation introduces an additional risk of a serious hemorrhage, emphasizing the importance of anticoagulation monitoring and the hemostatic balance.^3^, ^5^, ^6^, ^7^, ^8^, ^9^, ^10^, ^11^

Unfractionated heparin (UFH) is the most frequently used anticoagulant during ECMO, often regarded as the standard of care. Its anticoagulant properties are attributed to its ability to enhance the activity of antithrombin, downregulating thrombin and factor Xa.^3^, ^12^ Alongside UFH, heparin‐coated circuits are employed with the goal of minimizing the requirements for systemic anticoagulation.^13^ The primary mechanism of action involves heparin binding to the circuit surfaces, mimicking the antithrombogenic effects of heparan on the endothelium. These surfaces release minimal amounts of heparin into the blood, resulting in negligible systemic effects.^13^ Due to the inherent limitations of UFH, systemic anticoagulation is increasingly achieved using alternative methods, such as direct thrombin inhibitors (e.g., argatroban, bivalirudin, etc.), direct and indirect factor Xa inhibitors (e.g., rivaroxaban, apixaban, and edoxaban), heparinoids (e.g., danaparoid), and other novel anticoagulants (e.g., factor XIIa inhibitors, circuit releasing compounds, nitric oxide, etc.).^3^

The Extracorporeal Life Support Organization (ELSO) recommends the use of time‐guided anticoagulation tools (i.e., activated clotting time [ACT], activated prothrombin time [aPTT], clotting times generated from viscoelastic testing), or anti‐factor Xa assays for monitoring anticoagulation with UFH.^4^, ^14^, ^15^ Depending on the patient's hemostatic capacity, ELSO advocates anticoagulation goals of 1.5 to 2.5 times the patient's baseline for aPTT and 0.3–0.7 IU/mL for anti‐factor Xa assays.^14^ The International Society on Thrombosis and Haemostasis (ISTH) recommends anticoagulation using UFH, primarily monitored with anti‐factor Xa, with target levels of 0.3–0.5 IU/mL. Alternatively, ISTH suggests achieving an ACT of 180–220 s or an aPTT of 50–70 s.^16^

However, these anticoagulation goals have not been validated in controlled randomized trials or in critically ill patients undergoing ECMO. The most appropriate UFH monitoring tool in ECMO patients remains unclear, and strong evidence is lacking. Systematized evidence on the association of anti‐Xa levels with hemorrhagic events in patients receiving ECMO does not exist.

Therefore, we aimed to provide a comprehensive review and meta‐analysis investigating the relationship between measured anti‐factor Xa levels and hemorrhage, including its correlation with the UFH dose.

## 2 MATERIAL AND METHODS

A systematic literature review and meta‐analysis were conducted on studies investigating anticoagulation monitoring using anti‐factor Xa. The study protocol is registered in the international database of prospectively registered systematic reviews PROSPERO database—CRD42023448888, and the research was carried out in accordance with the PRISMA guidelines (Supporting Information S1: Table S1).^17^ The primary objective was to systematize evidence concerning the association between anti‐Xa levels and hemorrhage during ECMO support. Secondary endpoints included assessing the correlation between anti‐factor Xa levels and the UFH infusion rate, as well as the incidence of adverse events. All selected works comprised patients requiring ECMO, using anti‐factor Xa monitoring, and reporting the incidence of bleeding (Supporting Information S1: Table S2).

### 2.1 Search strategy

We performed a systematic search of literature in the PubMed and Scopus databases (up to August 2023). The search strategy involved combining terms related to ECMO support and anticoagulation monitoring (Supporting Information S1: Table S3). Ensuring the comprehensiveness of our search, we also reviewed the references of all included works. In case where full‐text works or detailed information on anti‐Xa levels were unavailable, authors of original work were contacted. Our inclusion criteria encompassed publications reporting on ECMO support, anti‐Xa anticoagulation monitoring, and bleeding. We excluded duplicate publications, articles reporting results from the same center, and systematic reviews or meta‐analyses. All data, search, and study restrictions are outlined in Supporting Information S1: Table S2. Two authors conducted screening of the literature (D. J., S. R.).

### 2.2 Data extraction

A summary of the extracted data is reported in Supporting Information S1: Table S4. To standardize the results of the analyzed works and facilitate comparison, the following calculations were performed: (A) conversion of percentages into original values, (B) the portion of male or female patients was calculated from the available information, (C) summation of outcomes of interest if provided for only one group, and (D) conversion of provided median values with ranges into means and standard deviations.^18^ All calculations were performed independently by two authors (D. J., S. R.).

### 2.3 Quality assessment of included studies

The methodological quality was assessed using the Newcastle‐Ottawa scale (NOS).^19^ Works scoring more than seven NOS stars were considered to be of good quality, those with at least five stars were considered fair, and those with fewer than five stars as low‐quality (Table 1).

**Table 1:** Characteristics of 26 included articles (*n* = 2293).

Author, country (study period)	Population age (years) male sex (%)	Sample size ECMO type (*n*)	ECMO duration (days)	Main ECMO indications	Main study aim	NOS
Al‐Jazairi et al., Saudi Arabia (–)	▪ Adult ▪ 42.9 ± 12.9 ▪ 15 (75)	▪ *n* = 20 ▪ VA: 13 ▪ VV: 7	7 (4–19)	▪ Cardiac arrest, failure to wean off in operating room, bridge to transplant, acute respiratory failure, and other.	▪ Correlation between anti‐Xa, ACT, and aPTT with UFH infusion.	Good
Arnouk et al., USA (2015–2017)	▪ Adult ▪ 56 (38–65) ▪ 26 (77)	▪ *n* = 34 ▪ VA: 21 ▪ VV: 13	3.9 (2.0–8.5)	▪ Cardiogenic shock, hypoxemic respiratory failure, ARDS, myocardial infarction, etc.	▪ Correlation anti‐Xa and aPTT with UFH infusion, ▪ Description of hemostatic adverse events.	Good
Bembea et al., USA (2008–2010)	▪ Pediatric ▪ 10 days (2 days– 10 years) ▪ 16 (44)	▪ *n* = 35 ▪ VA: 32 ▪ VV:3	7 (3–4)	▪ Respiratory failure, cardiac failure, eCPR, and sepsis.	▪ Correlation of ACT, anti‐Xa, and aPTT with UFH infusion.	Good
Delmas et al., France (2014–2015)	▪ Adult ▪ 54 (41.8–60) ▪ 76 (70)	▪ *n* = 109 ▪ VA: 77 ▪ VV: 32	5 (3–11)	▪ Cardiac arrest, cardiogenic shock, ARDS, etc.	▪ Measurement of USH activity with ACT and anti‐Xa assay.	Good
Descamps et al., France (multicentre) (2017–2019)	▪ Adult ▪ 51 (39–61) ▪ 80 (67)	▪ *n* = 121 ▪ VA: 77 ▪ VV: 44	6 (3–10)	▪ Refractory cardiac arrest, cardiogenic shock, ARDS, and intoxication.	▪ Association of anti‐Xa levels with hemorrhage.	Good
Deshpande et al., USA (2010–2016)	▪ Pediatric ▪ ‐ ▪ 73 (55)	▪ *n* = 133 ▪ VA: 92 ▪ VV:	‐	▪ CDH, ARDS, lower respiratory tract infection, sepsis, meconium aspiration syndrome, etc.	▪ Association of laboratory measurements with hemostatic complications, ▪ Correlation of different anticoagulation monitoring tools, ▪ UFH infusion correlation with laboratory parameters.	Good
Drop et al., Netherlands (2011‐2018)	▪ Pediatric ▪ 3.3 months (0.05–37.2) ▪ 33 (45)	▪ *n* = 73 ▪ VA: 43 ▪ VV: 30	5 (3–8)	▪ Respiratory, cardiac, and eCPR.	▪ Incidence of hemostatic adverse events, ▪ Association of adverse events with laboratory parameters.	Good
Feih et al., USA (2012–2018)	▪ Adult ▪ ‐ ▪ 46 (62)	▪ *n* = 74 ▪ VA: ‐ ▪ VV: ‐	‐	▪ Cardiogenic shock, respiratory failure, fail to wean from bypass.	▪ Identification of risk factors for hemostatic complications.	Good
Figueroa Villalba et al., USA (2015–2018)	▪ Pediatric ▪ ‐ ▪ 79 (52)	▪ *n* = 145 ▪ VA: 110 ▪ VV: 42	‐	▪ Respiratory failure, cardiogenic shock, postcardiac surgery, CDH, cardiac arrest, etc.	▪ Effect of change in monitoring from ACT to anti‐Xa.	Good
Henderson et al., USA (2013–2015)	▪ Pediatric ▪ 2.3 ± 2 ▪ 12 (40)	▪ *n* = 30 ▪ VA: 26 ▪ VV: 4	6.2 ± 1.6	▪ Congenital heart disease, cardiogenic shock, and cardiac arrest.	▪ Analysis of anticoagulation goals for predicting hemostatic complications	Good
Hohlfelder et al., USA (2013–2015)	▪ Pediatric ▪ 48 (24–68) ▪ 30 (63)	▪ *n* = 48 ▪ VA: 26 ▪ VV: 22	7 (2–84)	▪ Cardiogenic shock, respiratory failure and post‐transplantation.	▪ Correlation of UFH infusion with anticoagulation assays.	Good
Kessel et al., USA (2004–2013)	▪ Pediatric ▪ ‐ ▪ ‐	▪ *n* = 9 ▪ VA: 6 ▪ VV: 3		▪ ARDS, CDH, Pneumonia, etc.	▪ Correlation of UFH infusion with anticoagulation assays.	Good
Liveris et al., USA (2010–2012)	▪ Pediatric ▪ 0.83 years (1 day–13.9 years) ▪ 9 (53)	▪ *n* = 17 ▪ VA: 15 ▪ VV: 2	6 (1–34)	▪ Cardiogenic shock, postcardiotomy, sepsis, etc.	▪ Correlation of UFH infusion with anticoagulation assays.	Good
McMichael et al., USA (2012–2014)	▪ Pediatric ▪ ‐ ▪ ‐	▪ *n* = 69 ▪ VA: ‐ ▪ VV: ‐	‐	▪ Cardiogenic shock, respiratory failure, and other.	▪ Correlation among anticoagulation monitoring tools, ▪ Association of monitoring tools with survival and hemostatic complications.	Good
Meshulami et al., USA (2012–2014)	▪ Pediatric ▪ ‐ ▪ 300 (58)	▪ *n* = 514 ▪ VA: 436 ▪ VV: 78	‐	▪ Respiratory failure, cardiogenic shock, and eCPR.	▪ Association of hemostatic complications with anticoagulation monitoring.	Good
Moussa et al., France (2015–2019)	▪ Adult ▪ 55 ± 14 ▪ 183 (69)	▪ *n* = 265 ▪ VA: 265 ▪ VV: 0	7 (3–11)	▪ Myocardial infarction, postoperative low cardiac output, primary graft dysfunction, etc.	▪ Association between aPTT and anti‐Xa, ▪ Association of anticoagulation monitoring with complications.	Fair
Moynihan et al., Australia (2015–2016)	▪ Pediatric ▪ 17 days (2–764) ▪ 22 (69)	▪ *n* = 31 ▪ VA: 29 ▪ VV: 5	6 (3.6–9.2)	▪ Respiratory failure, post‐cardiac surgery, eCPR, etc.	▪ Correlation between anticoagulation monitoring tools and UFH infusion dose.	Good
Nankervis et al., USA (2004–2005)	▪ Pediatric ▪ ‐ ▪ ‐	▪ *n* = 12 ▪ VA: ‐ ▪ VV: ‐	11.4 ± 4.3	▪ CDH, sepsis, etc.	▪ Correlation between anticoagulation monitoring tools and UFH infusion dose.	Good
Nguyen et al., Vietnam (2019–2020)	▪ Adult ▪ 40 (32–50) ▪ ‐	▪ *n* = 37 ▪ VA: 23 ▪ VV: 13	7 (4–12)	▪ Acute myocarditis, ARDS, myocardial infarction, and anaphylaxis.	▪ Correlation between anticoagulation monitoring tools and UFH infusion dose.	Fair
Niebler et al., USA (2006–2016)	▪ Pediatric ▪ ‐ ▪ ‐	▪ *n* = 274 ▪ VA: ‐ ▪ VV: ‐	‐	▪ Cardiac and noncardiac surgery.	▪ Impact of anticoagulation monitoring on complications occurrence.	Good
O'Meara et al., USA (2013–2014)	▪ Pediatric ▪ 12.5 days (1 day–17 years) ▪ ‐	▪ *n* = 20 ▪ VA: 20 ▪ VV: 2	3.7 (2.4–7.6)	▪ Cardiorespiratory failure, eCPR, and pulmonary hypertension.	▪ Association of anti‐Xa assay monitoring with UFH management and oxygenator/circuit changes.	Fair
Perez Ortiz et al., Germany (2018–2019)	▪ Pediatric ▪ ‐ ▪ 16 (70)	▪ *n* = 23 ▪ VA: 26 ▪ VV: 0	10.3 (1–20)	▪ CDH.	▪ Correlations between ACT, anti‐Xa, aPTT, and ROTEM with UFH infusion.	Fair
Rabinowitz et al., USA (2018–2020)	▪ Pediatric ▪ 13.5 months (4–120 months) ▪ 18 (51)	▪ *n* = 35 ▪ VA: 21 ▪ VV: 13	6.4 (3.8–12.9)	▪ ARDS, postcardiotomy, cardiac arrest, etc.	▪ Correlation between anticoagulation monitoring tools and UFH infusion dose.	Good
Sleeper et al., USA (2015–2017)	▪ Pediatric ▪ 2.1 ± 3.7 ▪ 17 (43)	▪ *n* = 40 ▪ VA: ‐ ▪ VV: ‐	‐	▪ ‐	▪ Risk factors for bleeding during ECMO support (thromboelastography).	Fair
Sulkowski et al., USA (2008–2013)	▪ Pediatric ▪ ‐ ▪ 14 (24)	▪ *n* = 26 ▪ VA: ‐ ▪ VV: ‐	3.9 (2.6–7.4)	▪ CDH, sepsis, primary pulmonary hypertension, meconium aspiration, and cardiomyopathy.	▪ Correlation between anticoagulation monitoring tools and UFH infusion dose.	Fair
Yabrodi et al., USA (2013–2017)	▪ Pediatric ▪ 26 days (8–206) ▪ ‐	▪ *n* = 100 ▪ VA: ‐ ▪ VV: ‐	6.3 (0.7–15)	▪ Hypoplastic left heart syndrome, obstructive lesions of the left heart, etc.	▪ Correlation between anticoagulation monitoring tools and UFH infusion dose.	Fair

Abbreviations: ACT, activated clotting time; aPTT, activated partial thromboplastin time; ARDS, acute respiratory distress syndrome; CDH, congenital diaphragmatic hernia; ECMO, extracorporeal membrane oxygenation; ECPR, extracorporeal cardiopulmonary reanimation; NOS Newcastle‐Ottawa scale score; VA, venoarterial ECMO; VV, venovenous ECMO; UFH, unfractionated heparin.

### 2.4 Statistical analysis

Statistical assessments were completed using R software version 4.2.2 (“meta” and “metafor” packages; R Foundation for Statistical Computing, R Core Team 2022; Vienna, Austria) and SPSS (Version 22.0. Released 2013: IBM Corp.). Standardized mean differences (SMD) were utilized to compare patient groups, and these were calculated by pooling individual publication data using random or fixed effect models. We used the inverse variance methods with Fisher's *z* transformation for the pooled estimate of correlation coefficients. For ease of interpretation, back transformation (*z* to *r* transformation) to the original coefficients level was performed. Heterogeneity was analyzed by *τ* ^2^ and Cochran's *Q* test, and the results were reported with *I* ^2^. Using funnel plot and Egger's test, we assessed the publication bias. A significance level of 0.05 was applied.

## 3 RESULTS

### 3.1 Search results and included studies

**Figure 1:**
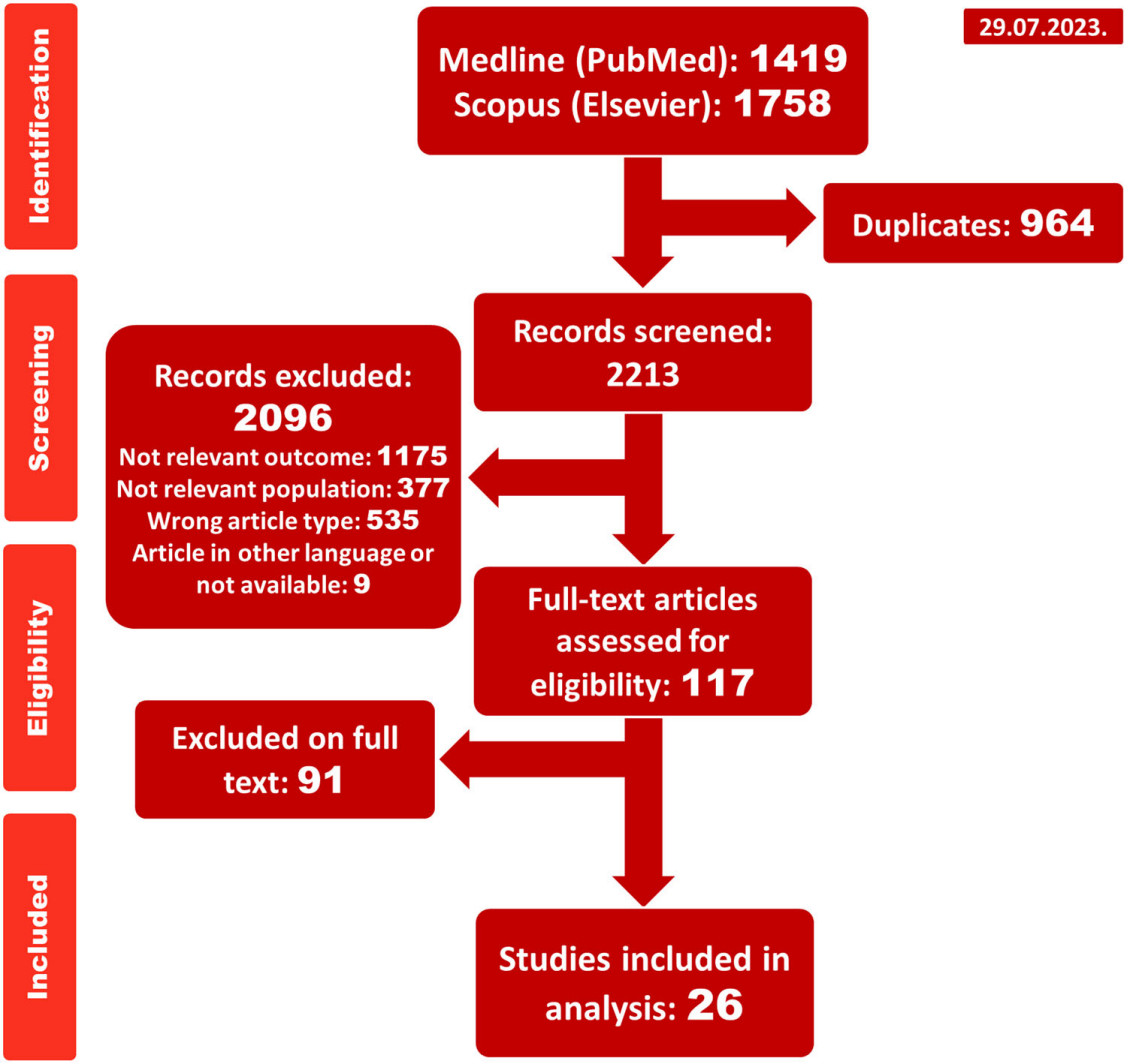
PRISMA flowchart. PRISMA, Preferred Reporting Items for Systematic Reviews and Meta‐analyses.

The initial search yielded 1419 and 1758 publications in PubMed and Scopus, respectively. Following the removal of duplicates, the titles and abstracts of 2213 works were screened. In the next step, 2130 works were excluded: 1175 due to irrelevant outcomes, 535 based on publication type, and 377 addressed irrelevant population (Figure 1). The excluded articles, along with the reason for exclusion, are detailed in Supporting Information S1: Table S5. Thereby, 117 articles underwent full‐text screening, resulting in the exclusion of an additional 91 articles. Finally, 26 studies met the inclusion criteria, with six studies reporting on the anti‐Xa levels being included in the meta‐analysis.

The characteristics of the included articles are available in Table 1. The analyzed publications represent the situation from the USA (*n* = 18), France (*n* = 3), and one each from the Netherlands, Germany, Vietnam, Saudi Arabia, and Australia. Twenty studies reported on both venovenous (VV) and venoarterial (VA) ECMO support, while four focused solely on VA‐ECMO. Additionally, 18 authors provided information on the correlation between UFH infusion and anti‐Xa blood levels.

### 3.2 Patient population and ECMO‐related outcomes

Between 2004 and 2020, a total of 2293 patients requiring ECMO support were included in the study. All authors used UFH as the primary anticoagulant, with target anti‐Xa levels ranging from 0.2 to 1.0 IU/mL, Table 1 and Supporting Information S1: Table S6.

Any form of bleeding (pooled 36%; 95% confidence interval [CI]: 28.1; 44.4) and major bleeding as defined by ELSO (pooled 36%; 95% CI: 21.9; 51.9) were the most prevalent adverse events (Table 2). Information on cerebral hemorrhage was reported in 11 studies, with a pooled rate of 10% (95% CI: 6.3; 14.9), while pulmonary and gastrointestinal bleeding were reported with pooled rate of 5% and 4%, respectively.

**Table 2:** ECMO‐related outcomes and adverse events (*n* = 26).

Outcome	Number of studies reporting data (events)	Pooled rate (95% CI)	*I* ^2^ (*p* Value)	Reported range (%)
Mortality				
In‐hospital mortality	15 (484)	47.0 (41.3; 52.8)	63% (<.01)	29.4–65.0
ICU mortality	3 (179)	45.4 (29.4; 62.5)	85% (<.01)	22.6–62.2
Death during ECMO	5 (121)	23.0 (13.3; 36.8)	71% (<.01)	15.4–38.9
Bleeding				
Any bleeding	17 (463)	35.9 (28.1; 44.4)	87% (<.01)	11.1–62.5
Major bleeding (ELSO)	6 (237)	35.5 (21.9; 51.9)	91% (<.01)	19.0–56.6
Cerebral hemorrhage	11 (114)	9.8 (6.3; 14.9)	75% (<.01)	2.3–20.6
Gastrointestinal bleeding	5 (21)	4.1 (2.7; 6.2)	12% (.34)	0.8–8.8
Pulmonary hemorrhage	6 (24)	4.5 (1.8; 10.5)	74% (<.01)	1.6–20.6
Thrombosis				
Any thrombosis	17 (375)	33.2 (24.7; 42.8)	89% (<.01)	6.6–90.0
Ischemic stroke	5 (69)	10.7 (6.6; 16.8)	61% (.04)	0.0–17.4
ECMO circuit and membrane clot	12 (176)	25.9 (17.1; 37.2)	87% (<.01)	5.0–58.8

Abbreviations: CI, confidence interval; ECMO, extracorporeal membrane oxygenation; ELSO, extracorporeal life support organization; ICU, intensive care unit.

Information regarding any type of thrombosis was provided in 17 studies, with a pooled rate of 33% (95% CI: 24.7; 42.8). The most common types of thrombosis reported were ECMO circuit and oxygenator membrane clotting (pooled 26%, 95% CI: 17.1; 37.2), followed by cerebral infarction (pooled rate: 11%; 95% CI: 6.6; 16.8), Table 2. Additionally, a total of 484 out of 991 patients (pooled: 47%; 95% CI: 41.3; 52.8) did not survive until discharge, while 23% of patients died during ECMO (Table 2 and Supporting Information S1: Table S7).

### 3.3 Hemorrhage and anti‐Xa

**Figure 2:**
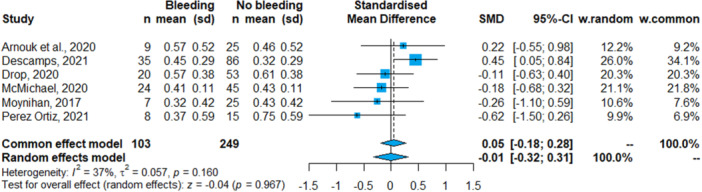
Forest plot: Average anti‐Xa values among patients with and without hemorrhagic event. CI, confidence interval; ECMO, extracorporeal membrane oxygenation; SMD, standardized mean differences.

The majority of the included works intended to describe the association between anti‐Xa and hemorrhage, as well as the correlation between anti‐Xa levels and the UFH dose. Seven studies (*n* = 391) reported the average anti‐Xa levels for patients with and without bleeding (Table 1). The meta‐analysis (six studies, *n* = 352) revealed no significant difference in the average values of anti‐Xa between patients with and without bleeding events (SMD = 0.05; 95% CI: −0.19; 0.28, *p* = .69); with heterogeneity of *I* ^2^ = 37% (95% CI: 0.0; 74.9, *Q* = 7.9, *τ* ^2^ = 0.06, *p* = .16) (Figure 2). One work did not report the variance of measured anti‐Xa levels and could not be included in the analysis.^20^ Finally, we did not identify the presence of publication bias (*p* = .160) (Supporting Information S1: Figure S1).

### 3.4 Sensitivity analysis

The sensitivity analysis identified Descamps et al. as a study that could significantly contribute to the heterogeneity observed. The results of the sensitivity analysis were consistent with the main analysis findings (Supporting Information S1: Figures S1.1–S1.4).

In the subgroup analysis based on pediatric and adult patient populations, the meta‐analysis of studies focusing on adult patients (two studies, *n* = 155) revealed higher anti‐Xa values in patients with bleeding events compared to those without (SMD = 0.56; 95% CI: 0.21; 0.92, *p* ≤ .001); with heterogeneity of *I* ^2^ = 0% (*Q* = 0.8, *τ* ^2^ = 0, *p* = .38) (Supporting Information S1: Figures S2.1–S2.3). Conversely, the meta‐analysis of studies focusing on pediatric patients (four studies, *n* = 197) did not identify a significant difference in the average values of anti‐Xa between patient groups (SMD = −0.21; 95% CI: −0.51; 0.10, *p* = .19); with heterogeneity of *I* ^2^ = 0% (*Q* = 0.12, *τ* ^2^ = 0, *p* = .99) (Supporting Information S1: Figures S3.1–S3.3).

### 3.5 Correlation of UFH dose with anti‐Xa

**Figure 3:**
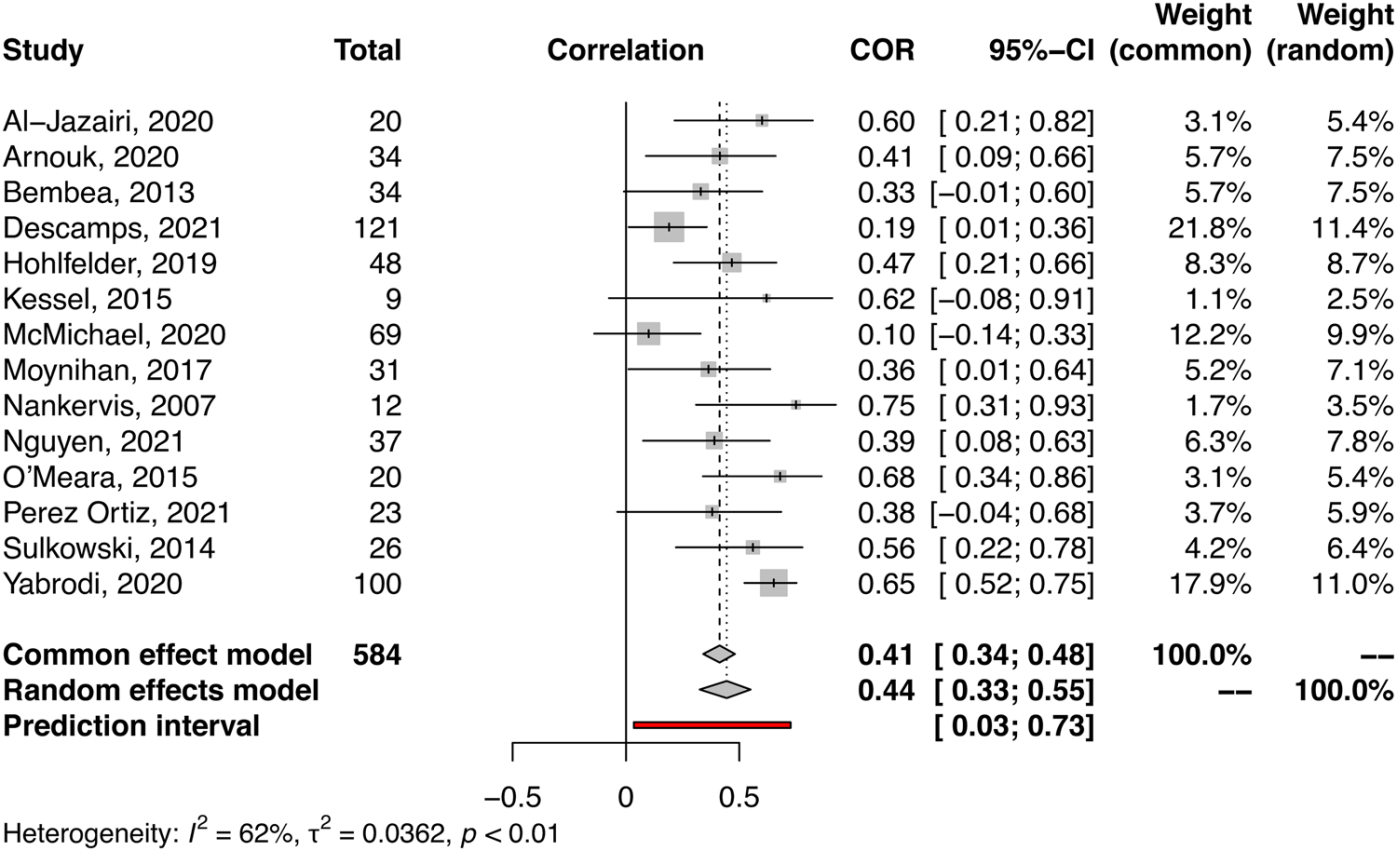
Forest plot: Meta‐analysis of correlation coefficients. CI, confidence interval; COR, correlation coefficient; ECMO, extracorporeal membrane oxygenation.

Eighteen studies (*n* = 878) provided data on the correlation between UFH infusion and anti‐Xa levels. Out of these, fourteen articles (*n* = 584) were eligible for inclusion in the meta‐analysis of correlation coefficients (Figure 3 and Supporting Information S1: Table S6). The correlation coefficients reported in the analyzed studies ranged from 0.1 to 0.75, all indicating a positive correlation between UFH infusion and anti‐Xa levels. The pooled estimate of correlation coefficients was 0.44 (95% CI: 0.33; 0.55, *p* < .001), with heterogeneity of *I* ^*2*^ = 62% (*τ* ^2^ = 0.02, *p* = .001) (Figure 3). Sensitivity analysis (excluding Yabordi et al.) reaffirmed the findings of the main analysis (Supporting Information S1: Figures S4–S5 and Table S8).

In the subgroup analysis based on pediatric and adult patient populations, four studies (*n* = 212) reported on the correlation of UFH infusion with anti‐Xa levels for adult patients and 10 (*n* = 372) for pediatric population. For adult patients, the pooled estimate of correlation coefficients was 0.35 (95% CI: 0.16; 0.52, *p* < .001), with heterogeneity of *I* ^2^ = 40% (*τ* ^2^ = 0.02, *p* = .17) (Supporting Information S1: Figure S2.3). In contrast, for pediatric patients, the pooled estimate of correlation coefficients was 0.48 (95% CI: 0.33; 0.61, *p* < .001), with heterogeneity of *I* ^2^ = 62% (*τ* ^2^ = 0.04, *p* < .01) (Supporting Information S1: Figure S3.3).

## 4 DISCUSSION

The present work evaluated use of anti‐Xa‐guided anticoagulation monitoring in critically ill patients requiring ECMO support. Our analysis encompassed 26 articles with 2293 patients, representing the most extensive analysis to date of anti‐Xa‐guided anticoagulation monitoring. There was no significant difference in anti‐Xa levels between patients with and without hemorrhagic events. However, for the first time we report systematized evidence on a moderate correlation between UFH dose and anti‐Xa levels in ECMO‐supported patients.

### 4.1 Anticoagulation monitoring during ECMO support

The optimal anticoagulation in critically ill is challenging, especially in those needing ECMO support. Balancing anticoagulation to avoid thromboembolic complications against hemorrhage in this patient population remains difficult. Existing evidence on anticoagulation monitoring primarily derives from retrospective and observational works, with significant diversity between centers and the optimal anticoagulation remaining uncertain.^21^, ^22^, ^23^ The most recent edition of the ELSO Red Book highlighted the complexity of anticoagulation and its monitoring, acknowledging the scarcity of evidence and providing limited recommendations in this regard.^24^

The current anticoagulation goals lack validation in ECMO patients and are derived from a prospective conducted in adults back in 1972.^3^, ^15^, ^25^ According to the ISTH, the recommended anti‐factor Xa anticoagulation targets (0.3–0.5 U/mL) are slightly lower than those of ELSO (0.3–0.7 U/mL).^16^ These recommendations are supported by findings from three retrospective and two randomized controlled trials. Authors of mentioned works have reported an increased rate of major hemorrhage and hemorrhage‐related death among patients with higher anticoagulation targets (180–220 vs. 140–160 s), with no significant disparity observed in the frequency of oxygenator replacements or major thrombotic events.^26^ Another trial found no discernible difference in outcomes when comparing low‐ and high‐dose UFH anticoagulation based on ACT,^27^ or when comparing restricted or conventional anti‐factor Xa protocols (0.3–0.7 IU/mL vs. 0.2–0.5 IU/mL).^28^ However, all analyzed studies are constrained by small patient populations, indicating the need for further research.

However, the altered hemostatic capacity of ECMO patients, particularly in case of hyperinflammation, may limit the UFH effect by increasing its binding to acute phase proteins.^3^ In such circumstances, the use of time‐guided monitoring strategies may be biased with the limitations of the available tests. Optimal use of aPTT requires a linear relationship between aPTT and UFH dose, which is not the case in severely ill, where aPTT could be altered by the existing hemostatic changes (i.e., coagulopathy‐associated consumption of coagulation factors, elevated C‐reactive protein, lupus inhibitor, fibrinogen, factor VIII, etc.). Moreover, deficiencies in the contact pathway (kallikrein‐kinin) or factor XII may result in a spontaneous aPTT prolongation.^29^, ^30^, ^31^ Finally, the poor correlation between time‐based methods (both aPTT and ACT) and UFH dose reduces the applicability of these assays, despite their wide availability, low cost, and short turnaround time.^3^, ^32^ Thus, the introduction of alternative methods or multimodal approach is becoming more interesting for clinicians, but the evidence from large trials is still missing.^3^, ^15^, ^23^

In the analyzed articles, we observed different anticoagulation targets, ranging from 0.2 to 1.0 for anti‐Xa, 45 to 100, and 160 to 225 s for aPTT and ACT, respectively. Interestingly, there was no association between higher or lower anticoagulation targets and factors such as patient population, indication for ECMO support, or type of support. This finding is surprising considering that coagulation processes may differ between pediatric and adult population; or between patients with acute respiratory distress syndrome and those with cardiogenic shock. However, this may be attributed to the current diversity in protocols among different centers and the lack of standardization.

Our meta‐analysis did not reveal a significant difference in anti‐Xa values between ECMO patients with and without hemorrhage. This could be attributed to the limited evidence available on anti‐Xa‐guided anticoagulation monitoring, or the quality of reporting. Among the 26 studies included, only seven authors reported anti‐Xa values for patients with and without bleeding. Additionally, in four studies, anti‐Xa levels were slightly higher in patients without hemorrhage compared with those with hemorrhage. This could suggest that clinicians may adopt a more cautious approach to anticoagulation in patients who are clinically identified as having an increased risk of hemorrhage, potentially leading to contradictory findings. Clinicians might strive for lower anti‐Xa goals to mitigate bleeding risk while still maintaining anticoagulation within the predefined range. This might be particularly relevant in the pediatric population, as all studies reported lower average anti‐Xa levels in patients with bleeding. Conversely, only two studies provided average anti‐Xa levels for adult patients, both of which reported higher average anti‐Xa values in patients experiencing hemorrhage events.

An additional factor that may explain our findings across all studies is the variability in the timing of anti‐Xa measurement, which is often not clearly specified or standardized in retrospective studies. Anti‐Xa levels can be reported as an average over the whole ECMO period, as the highest/lowest value shortly before the event, or even more complex in case of multiple bleeding events. The only way to overcome this key limitation are prospective or randomized trials, where the time point of adverse events and laboratory measurements could be exactly examined.

Moreover, it's important to consider the intrinsic limitations of the anti‐Xa assay, which could act as a confounding factor in the contradictory findings observed. Factors such as high plasma‐free hemoglobin, hyperbilirubinemia, or hyperlipidaemia can lead to falsely low anti‐Xa values. Both hyperbilirubinemia and increased free hemoglobin (due to hemolysis) are frequently seen in patients receiving ECMO, potentially limiting its application. Moreover, the anti‐Xa is a plasma‐based assay, excluding the role of platelet and fibrinogen in the formation of clot. Therefore, underestimation of the platelets/fibrinogen role or false low anti‐Xa levels may lead to increased UFH dose and unintentional over‐anticoagulation.^14^, ^24^, ^33^ Despite these limitations, anti‐Xa may still offer advantages over traditional assays like aPTT, particularly in situations involving elevated C‐reactive protein or the presence of lupus anticoagulant.

### 4.2 Correlation of UFH dose with anti‐Xa

Fourteen articles were included in the meta‐analysis of correlation coefficients, all of which demonstrated a positive correlation between anti‐Xa and the UFH dose. Despite existing heterogeneity, we observed a moderate correlation between UFH and anti‐Xa. These findings align with those reported by Padhya et al., who observed a strong correlation based on the analysis of four studies.^34^ The authors highlighted the importance of anti‐Xa monitoring as the only laboratory test (compared to ACT and aPTT) having strong correlation with the UFH dose, which is the most suitable tool for anticoagulation monitoring during ECMO. Given the data from analyzed studies, we support the measurement of anti‐Xa activity to monitor UFH in patients needing ECMO support.

### 4.3 Adverse events and mortality

The current evidence comprises works with small patient populations and a wide range of reported complications, lacking standardization in reporting. We identified bleeding as the most frequent complication, which is in line with the available literature.^9^, ^35^, ^36^ However, complications such as acute kidney injury and the need for renal replacement therapy, commonly associated with ECMO support, were inconsistently reported across studies, limiting detailed analysis. Moreover, data on other adverse events varied in reporting and frequency, possibly influenced by a selection bias favouring works focusing on anticoagulation monitoring and hemorrhage.

Thromboembolic complications, including ECMO circuit and oxygenator clot formation, were reported in 17 out of 26 studies, indicating a relatively high incidence compared to previous reports.^9^, ^35^, ^36^ However, the true incidence of thrombosis remains uncertain due to potential underestimation in retrospective and observational studies.^37^, ^38^

Finally, we estimated a pooled in‐hospital mortality of 47%, consistent with the most recent ELSO report (46%, based on 198 623 ECMO runs).^39^

### 4.4 Future development and outlook

Further research on patient‐related factors, potential adverse events predictors, ECMO pumps and circuits, and surgical techniques is warranted. This holds especially true in the field of existing and emerging anticoagulation strategies and monitoring approaches. Other anticoagulation modalities (i.e., low‐molecular‐weight heparin, antibodies targeting factors XI and XII, small‐molecule activated factor Xia inhibitors (EP‐7041), etc.) or ECMO support without anticoagulation, earlier an unthinkable method are subject of intensive research.^40^, ^41^, ^42^, ^43^, ^44^, ^45^, ^46^, ^47^, ^48^, ^49^ Furthermore, the data on the UFH monitoring is slowly occurring, particularly in regard to the newer methods like anti‐Xa assays, viscoelastic tests or multimodal approaches.^7^, ^10^, ^32^, ^33^, ^50^, ^51^ Despite the promising results and ongoing studies, we cannot estimate the coagulation system in vivo or to utilize continuous monitoring, like for blood gas or hemoglobin monitoring.^52^

On the other hand, the use of direct thrombin inhibitors (i.e., argatroban and bivalirudin) is gaining popularity, extending its indication profile beyond the heparin induced thrombocytopenia (HIT) and heparin‐resistance.^3^ They bond directly to free circulating and also fibrin bounded thrombin (unlike UFH). Moreover, they are antithrombin independent and have more predictable pharmacokinetics.^53^ Further research on their benefits and limitations, including monitoring is warranted.

Finally, potentially modifiable risk factors and predictors of bleeding should be early recognized and promptly managed. However, due to the lack of guidelines on reporting criteria for patients undergoing ECMO support, there is considerable heterogeneity in how these events are reported across studies. As a result, systematizing risk factor assessment and prediction remains complex. However, as the increasing number of authors use the ELSO hemorrhage definition, reporting on major bleeding events is becoming more standardized and comparable across studies.

### 4.5 Strengths and limitations

The strengths of this work include 26 publications involving 2293 patients, and a good methodological quality. Moreover, all works from the same centers were excluded, controlling for potential patient overlap; and we report on a systematized search of large databases according to the PRISMA checklist.

Nonetheless, our work has some limitations. Publication and retrieval bias cannot be excluded. It could be that the studies are not published or available in the searched databases. The quality of reported evidence is as strong as the studies analyzed, given their predominantly retrospective type. Moreover, authors reported heterogeneous on adverse events, missing any systematization, and making their comparison demanding. The ELSO definition of hemorrhage was utilized by less than half of the authors, and the assortment of research aims led to variety in outcomes, and the data on hemorrhagic events subgroups was only sporadically available.

Furthermore, for comparison reasons and using a validated tool, we converted reported median values into mean with standard deviations, as we did not have access to the original patient data used in the studies. This should lead to a rather insignificant and controlled alterations in results.

Heterogeneity is an important limitation of retrospective studies, and some results of our analysis should be interpreted cautiously.

Finally, despite the fact that our robust search criteria resulted in the final analysis of 26 articles, the available information on the anti‐Xa‐guided anticoagulation and hemorrhage is limited and slowly emerging. However, we were still able to systematize the information related to VA and VV ECMO support and to conduct the meta‐analysis.

## 5 CONCLUSION

Despite significant advancements in extracorporeal life support technology, the most appropriate anticoagulation monitoring tool during ECMO is uncertain. Our analysis did not reveal a significant difference in anti‐Xa levels in patients with and without hemorrhagic events. However, we found a moderate correlation between anti‐Xa and the UFH dose, supporting its utilization in monitoring UFH anticoagulation. Given the limitations of time‐guided monitoring methods, the role of anti‐Xa is promising, and further research is warranted.

## AUTHOR CONTRIBUTIONS

*Conceptualization*: Sasa Rajsic, Zoran Bukumiric, and Robert Breitkopf. *Data curation*: Sasa Rajsic, Zoran Bukumiric, Robert Breitkopf, and Dragana Jadzic. *Formal analysis*: Sasa Rajsic and Zoran Bukumiric. *Investigation*: Sasa Rajsic, Zoran Bukumiric, Christine Eckhardt, Christoph Oberleitner, Dragana Jadzic and Benedikt Treml. *Methodology*: Sasa Rajsic and Zoran Bukumiric. *Project administration*: Sasa Rajsic and Robert Breitkopf. *Resources*: Sasa Rajsic and Christine Eckhardt. *Software*: Sasa Rajsic and Zoran Bukumiric. *Supervision*: Sasa Rajsic and Robert Breitkopf. *Validation*: Sasa Rajsic, Robert Breitkopf, Zoran Bukumiric, and Nicole Innerhofer. *Visualization*: Sasa Rajsic, Zoran Bukumiric, and Robert Breitkopf. *Writing—original draft*: Sasa Rajsic, Robert Breitkopf, Dragana Jadzic, Zoran Bukumiric, Christine Eckhardt, Nicole Innerhofer, Christoph Oberleitner, and Benedikt Treml. *Writing—review and editing*: Sasa Rajsic, Robert Breitkopf, Dragana Jadzic, Zoran Bukumiric, Christine Eckhardt, Nicole Innerhofer, Christoph Oberleitner, and Benedikt Treml. All authors have read and agreed to the published version of the manuscript.

## CONFLICT OF INTEREST STATEMENT

The authors declare no conflict of interest.

## Supporting information

Supporting information.

## Data Availability

All data generated or analyzed during this study are included in this published article and its supplementary information files. The additional datasets used and analyzed during the current study are available from the corresponding author on reasonable request.
