# Supplementary material for: Association of anti‐factor Xa‐guided anticoagulation with hemorrhage during ECMO support: A systematic review and meta‐analysis
Source: Clin Cardiol. 2024 May 2;47(5):e24273. doi: 10.1002/clc.24273 (PMC11063723; doi:10.1002/clc.24273)
Supplement: Supplementary file 1 — Supporting information. [file CLC-47-e24273-s001.docx]

**Supplementary material**

**Association of anti-Xa-guided anticoagulation with hemorrhage during ECMO support:**

**A systematic review and meta-analysis**

# Contents

**Table S1.** Preferred Reporting Items for Systematic review and Meta-Analysis (PRISMA) 2020 checklist

**Table S2.** PICOS criteria for inclusion and exclusion of publications

**Table S3.** Search strategy

**Table S4.** Detailed information on the data extraction

**Table S5.** Main excluded studies

**Table S6.** Anticoagulation monitoring and outcomes of 26 included articles (n = 2293)

**Table S7.** Reported adverse events associated with ECMO in the included studies (n = 16)

**Figure S1.** Funnel plot of publication bias

**Figure S1.1.** Influence analysis: Baujat plot

**Figure S1.2.** Influence analysis: Studies sorted by the effect size.

**Figure S1.3.** Influence analysis: Studies sorted by the *I^2^*.

**Figure S1.4.** Influence analysis.

**Figure S2.1.** Forest plot: Average anti-Xa values among adult patients with and without haemorrhagic event. ECMO extracorporeal membrane oxygenation.

**Figure S2.2.** Funnel plot of publication bias (studies including adult population).

**Figure S2.3.** Forest plot: Meta-analysis of correlation coefficients (studies including adult population).

**Figure S3.1.** Forest plot: Average anti-Xa values among paediatric patients with and without haemorrhagic event.

**Figure S3.2.** Funnel plot of publication bias (studies including pediatric population).

**Figure S3.3.** Forest plot: Meta-analysis of correlation coefficients (studies including paediatric population).

**Figure S4.** Meta-analysis of correlation coefficients: Funnel and Baujat plot of publication bias

**Figure S5.** Meta-analysis of correlation coefficients: Influence analysis

**Table S8.** Meta-analysis of correlation coefficients: Influence analysis of included studies

# Table S1. Preferred Reporting Items for Systematic review and Meta-Analysis (PRISMA) 2020 checklist

| **Section and Topic** | **Item #** | **Checklist item** | **Location where item is reported** |
| --- | --- | --- | --- |
| **TITLE** | | |  |
| Title | 1 | Identify the report as a systematic review. | Page 1 |
| **ABSTRACT** | | |  |
| Abstract | 2 | See the PRISMA 2020 for Abstracts checklist. | Page 1 |
| **INTRODUCTION** | | |  |
| Rationale | 3 | Describe the rationale for the review in the context of existing knowledge. | Page 3-4 |
| Objectives | 4 | Provide an explicit statement of the objective(s) or question(s) the review addresses. | Page 4 |
| **METHODS** | | |  |
| Eligibility criteria | 5 | Specify the inclusion and exclusion criteria for the review and how studies were grouped for the syntheses. | Page 4-6 |
| Information sources | 6 | Specify all databases, registers, websites, organisations, reference lists and other sources searched or consulted to identify studies. Specify the date when each source was last searched or consulted. | Page 4-6 |
| Search strategy | 7 | Present the full search strategies for all databases, registers and websites, including any filters and limits used. | Page 4-6, Supplementary |
| Selection process | 8 | Specify the methods used to decide whether a study met the inclusion criteria of the review, including how many reviewers screened each record and each report retrieved, whether they worked independently, and if applicable, details of automation tools used in the process. | Page 5-6 |
| Data collection process | 9 | Specify the methods used to collect data from reports, including how many reviewers collected data from each report, whether they worked independently, any processes for obtaining or confirming data from study investigators, and if applicable, details of automation tools used in the process. | Page 5-6 |
| Data items | 10a | List and define all outcomes for which data were sought. Specify whether all results that were compatible with each outcome domain in each study were sought (e.g. for all measures, time points, analyses), and if not, the methods used to decide which results to collect. | Page 5-6, Supplementary |
|  | 10b | List and define all other variables for which data were sought (e.g. participant and intervention characteristics, funding sources). Describe any assumptions made about any missing or unclear information. | Page 5-6, Supplementary |
| Study risk of bias assessment | 11 | Specify the methods used to assess risk of bias in the included studies, including details of the tool(s) used, how many reviewers assessed each study and whether they worked independently, and if applicable, details of automation tools used in the process. | Page 6 |
| Effect measures | 12 | Specify for each outcome the effect measure(s) (e.g. risk ratio, mean difference) used in the synthesis or presentation of results. | Page 6 |
| Synthesis methods | 13a | Describe the processes used to decide which studies were eligible for each synthesis (e.g. tabulating the study intervention characteristics and comparing against the planned groups for each synthesis (item #5)). | Page 4-6 |
|  | 13b | Describe any methods required to prepare the data for presentation or synthesis, such as handling of missing summary statistics, or data conversions. | Page 4-6 |
|  | 13c | Describe any methods used to tabulate or visually display results of individual studies and syntheses. | Page 4-6, Supplementary |
|  | 13d | Describe any methods used to synthesize results and provide a rationale for the choice(s). If meta-analysis was performed, describe the model(s), method(s) to identify the presence and extent of statistical heterogeneity, and software package(s) used. | Page 4-6 |
|  | 13e | Describe any methods used to explore possible causes of heterogeneity among study results (e.g. subgroup analysis, meta-regression). | Page 6, Supplementary |
|  | 13f | Describe any sensitivity analyses conducted to assess robustness of the synthesized results. | Page 6 |
| Reporting bias assessment | 14 | Describe any methods used to assess risk of bias due to missing results in a synthesis (arising from reporting biases). | Page 6 |
| Certainty assessment | 15 | Describe any methods used to assess certainty (or confidence) in the body of evidence for an outcome. | Page 6 |
| **RESULTS** | | |  |
| Study selection | 16a | Describe the results of the search and selection process, from the number of records identified in the search to the number of studies included in the review, ideally using a flow diagram. | Page 6-7 |
|  | 16b | Cite studies that might appear to meet the inclusion criteria, but which were excluded, and explain why they were excluded. | Supplementary |
| Study characteristics | 17 | Cite each included study and present its characteristics. | Page 24-28 |
| Risk of bias in studies | 18 | Present assessments of risk of bias for each included study. | Page 24-28 |
| Results of individual studies | 19 | For all outcomes, present, for each study: (a) summary statistics for each group (where appropriate) and (b) an effect estimate and its precision (e.g. confidence/credible interval), ideally using structured tables or plots. | Page 24-33, Supplementary |
| Results of syntheses | 20a | For each synthesis, briefly summarise the characteristics and risk of bias among contributing studies. | Page 24-33, Supplementary |
|  | 20b | Present results of all statistical syntheses conducted. If meta-analysis was done, present for each the summary estimate and its precision (e.g. confidence/credible interval) and measures of statistical heterogeneity. If comparing groups, describe the direction of the effect. | Page 8-13, Supplementary |
|  | 20c | Present results of all investigations of possible causes of heterogeneity among study results. | Page 7-13 Supplementary |
|  | 20d | Present results of all sensitivity analyses conducted to assess the robustness of the synthesized results. | Supplementary |
| Reporting biases | 21 | Present assessments of risk of bias due to missing results (arising from reporting biases) for each synthesis assessed. | Page 7-13 |
| Certainty of evidence | 22 | Present assessments of certainty (or confidence) in the body of evidence for each outcome assessed. | Page 7-13 |
| **DISCUSSION** | | |  |
| Discussion | 23a | Provide a general interpretation of the results in the context of other evidence. | Page 13-18 |
|  | 23b | Discuss any limitations of the evidence included in the review. | Page 18-19 |
|  | 23c | Discuss any limitations of the review processes used. | Page 18-19 |
|  | 23d | Discuss implications of the results for practice, policy, and future research. | Page 14-21 |
| **OTHER INFORMATION** | | |  |
| Registration and protocol | 24a | Provide registration information for the review, including register name and registration number, or state that the review was not registered. | Page 4 |
|  | 24b | Indicate where the review protocol can be accessed, or state that a protocol was not prepared. | Page 4 |
|  | 24c | Describe and explain any amendments to information provided at registration or in the protocol. | Page 4 |
| Support | 25 | Describe sources of financial or non-financial support for the review, and the role of the funders or sponsors in the review. | Page 21 |
| Competing interests | 26 | Declare any competing interests of review authors. | Page 21 |
| Availability of data, code and other materials | 27 | Report which of the following are publicly available and where they can be found: template data collection forms; data extracted from included studies; data used for all analyses; analytic code; any other materials used in the review. | Page 21 |

*From: Page MJ, McKenzie JE, Bossuyt PM, Boutron I, Hoffmann TC, Mulrow CD, et al. The PRISMA 2020 statement: an updated guideline for reporting systematic reviews. BMJ 2021;372:n71. doi: 10.1136/bmj.n71.*

# Table S2. PICOS criteria for inclusion and exclusion of publications

| **Parameter** | **Inclusion criteria** | **Exclusion criteria** |
| --- | --- | --- |
| Population | - Patients receiving ECMO support | - Patients receiving other mechanical life support  - Potential patient overlapping (i.e., studies from the same center, register studies overlapping with submitting center) |
| Intervention | - ECMO  - Anticoagulation with UFH  - Anticoagulation monitoring using anti-Xa  - Correlation of UFH dose with anti-Xa | - Studies not reporting on anti-Xa anticoagulation monitoring  - Other or no anticoagulation |
| Comparator | None | None |
| Outcomes | Primary:  - Anticoagulation monitoring using anti-Xa  - The rate of hemorrhagic events  Secondary:  - Correlation of UFH infusion with the anti-Xa levels,  - Demographic and clinical characteristics of patients receiving ECMO support,  - Association of anti-Xa monitoring with bleeding,  - Reported adverse events,  - In-hospital mortality | - |
| Study design | - Randomized trials,  - Prospective and retrospective cohort studies,  - Grey literature | - Meta-analyses,  - Systematic reviews,  - Conference abstracts,  - Case reports,  - Short reports,  - Letters |
| *PICOS* patients/population, intervention, comparator, outcomes, study design, *ECMO* extracorporeal membrane oxygenation, *UFH* unfractionated heparin | | |

# Table S3. Search strategy

| **Database: PubMed** |
| --- |
| Search date: 29.07.2023.  Total number of records: 1419  Code: ((((extracorporeal membrane oxygenation[MeSH Terms]) OR (ECMO)) OR (extracorporeal life support)) OR (ECLS)) AND ((((((((anticoagulation monitoring) OR (anticoagulation)) OR ("Blood Coagulation Tests"[Mesh])) OR (Activated clotting time)) OR (ACT)) OR (anti Xa)) OR (anti-factor Xa))) Filters: Humans |
| **Database: Scopus** |
| Search date: 29.07.2023.  Total number of records: 1758  Code: (TITLE-ABS-KEY ("extracorporeal membrane oxygenation" OR ECMO OR "extracorporeal life support" OR ECLS AND "anticoagulation monitoring" OR anticoagulation OR ACT OR "Activated clotting time" OR "anti Xa" OR "anti-factor Xa")) |

# Table S4. Detailed information on the data extraction

| **Characteristics** | **Description** |
| --- | --- |
| Study characteristics | Author, study design, publication year, number of patients, recruitment period, institution and country, type of ECMO support, and main aim. |
| Patient and ECMO characteristics | Sex (male/female), age, SAPS II and III, the APACHE II, the SOFA score, cannulation type, and ECMO support duration. |
| Adverse events | Reported hemorrhagic events, major hemorrhagic events (ELSO), cannulation and surgical site bleeding, gastrointestinal tract bleeding, pulmonary bleeding, intracerebral hemorrhage; thrombotic events, ischemic stroke, venous thrombosis, deep vein thrombosis, limb ischemia, ECMO circuit and membrane clot; renal replacement therapy and acute kidney injury; and sepsis. |
| Mortality | Mortality during ECMO, critical care unit and hospital stay. |
| Anticoagulation regime | Type of anticoagulation, information on monitoring, goal anti-Xa range, use of bolus during cannulation, use of continuous infusion, the use of UFH coating, and the authors conclusion on association of anticoagulation monitoring with bleeding. |
| ECMO-related technical characteristics | ECMO machine used, pump type, and equipment-related adverse events. |

*ECMO* extracorporeal membrane oxygenation, *ELSO* extracorporeal life support organization, *UFH* unfractionated heparin, *APACHE* *II* acute physiology and chronic health evaluation II, *SAPS* simplified acute physiology score, *SOFA* sequential organ failure assessment.

# Table S5. Main excluded studies

| **Study (author and year)** | **State** | **Reason for exclusion** |
| --- | --- | --- |
| Alkazemi et al. 2021 | USA | No outcome of interest |
| Anton-Martin et al. 2017 | USA | Risk of patient overlap |
| Aubron et al. 2019 | Australia | No outcome of interest |
| Burton et al. 2021 | USA | Risk of patient overlap |
| Haftmann et al. 2023 | USA | ECMO patients excluded |
| Irby et al. 2014 | USA | No outcome of interest |
| Kulig et al. 2021 | USA | No outcome of interest |
| Northam et al. 2021 | USA | No outcome of interest |
| Panigada et al. 2016 | Italy | No outcome of interest |
| Rama et al. 2021 | USA | No outcome of interest |
| Rhoades et al. 2021 | USA | Publication type |
| Vo et al. 2022 | USA | No outcome of interest |
| Zaaqoq et al. 2022 | USA | No outcome of interest |

*ECMO* extracorporeal membrane oxygenation

# Table **S6**. Anticoagulation monitoring and outcomes of 26 included articles (n = 2293)

| **Author** | **Anticoagulation goal**  **ACT (s)**  **aPTT (s)**  **anti-Xa (IU/mL)** | **Samplings per day** | **Anti-Xa level (bleeding)^a^** | **Anti-Xa level**  **(no bleeding)^a^** | **Correlation with UFH infusion^b^** | **Association of monitoring with bleeding** |
| --- | --- | --- | --- | --- | --- | --- |
| Al-Jazairi et al. | ACT: 180-220 or 160-180  aPTT: -  anti-Xa: 0.3-0.7 or 02-0.4 | ▪ - | - | - | ▪ Strong: r = 0.6 (p < 0.05)  ▪ Anti-Xa may provide a better prediction of anticoagulation effect in relation to UFH dose compared to other tests. | ▪ - |
| Arnouk et al. | ACT: -  aPTT: -  anti-Xa: 0.3-0.7 | ▪ - | 0.56  (0.28-0.88) | 0.52  (0.17-0.68) | ▪ Moderate: *r* = 0.414 (p < 0.001)  ▪ Anti-Xa assay correlated better with weight based UFH dose compared to aPTT. | ▪ No: No difference in the maximum anti-Xa values between groups (p = 0.368). |
| Bembea et al. | ACT: 180-220  aPTT: -  anti-Xa: - | ▪ 3 | - | - | ▪ Weak: r = 0.33  ▪ Anti-Xa had a direct relation to UFH dose. | ▪ Yes: The percentage of discordant ACT to anti-Xa values that could indicate excessive anticoagulation (high anti-Xa and low ACT) was significantly higher in patients with bleeding. |
| Delmas et al. | ACT: 180-220  aPTT: -  anti-Xa: 0.2-0.4 | ▪ 1 | - | - | ▪ Weak  ▪ ACT seems more poorly correlated with UFH infusion compared to anti-Xa, justifying the preferential use of the later. | ▪ - |
| Descamps et al. | ACT: -  aPTT: -  anti-Xa: 0.2-0.7 | ▪ 6 | 0.38  (0.29-0.67) | 0.33  (0.22-0.42) | ▪ Weak: *r* = 0.190 (p = 0.064) | ▪ Yes: Mean anti-Xa is an independent risk factor for haemorrhage (HR = 16.10, 95% CI 3.9-65.8, p < 0.001).  ▪ The cut-off value is calculated to be 0.46 IU/mL (AUROC: 0.65, 95% CI 0.6-0.7, p = 0.018). |
| Deshpande et al. | ACT: -  aPTT: -  anti-Xa: 0.3-0.7 | ▪ - | - | - | ▪ Weak: R^2=^ 0.037, 95% CI 0.023-0.053 | ▪ No: There is no significant association between anti-Xa level, aPTT, ACT, or heparin dose with bleeding. |
| Drop et al. | ACT: -  aPTT: 50-70 or 60-85  anti-Xa: 0.3-0.7 or 0.5-1.0 | ▪ 4 | 0.59  (0.325-0.795)^c^ | 0.61  (0.44-0.79)^c^ | ▪ - | ▪ No: No difference in anti-Xa in patients with or without bleeding (0.59 IU/mL vs. 0.61 IU/mL, p = 0.51). |
| Feih et al. | ACT: 160-220  aPTT: -  anti-Xa: 0.21-0.35 or 0.3-0.7 | ▪ - | - | - | ▪ - | ▪ Yes: Protocol using Anti-Xa was associated with a lower hazard of major haemorrhage (HR = 0.388, IQR 0.22, 0.7, p = 0.002).  ▪ Anti-Xa targeting 0.21-0.35 IU/mL is associated with lower rates of major haemorrhage. |
| Figueroa Villalba et al. | ACT: 180-220  aPTT: -  anti-Xa: 0.2-0.4 | ▪ 4 | - | - | ▪ UFH therapy is more stable under anti-Xa monitoring. | ▪ Yes: The number of patients with reported bleeding decreased from 69% to 51% (p = 0.03). |
| Henderson et al. | ACT: -  aPTT: -  anti-Xa: 0.2-0.7 | ▪ 4 | - | - | ▪ - | ▪ No: None of the anticoagulation monitoring tools (ACT, aPTT, anti-Xa, TEG) was associated with bleeding.  ▪ No optimal anti-Xa cut-off for bleeding prediction. |
| Hohlfelder et al. | ACT: 180-220  aPTT: 60-80  anti-Xa: 0.3-0.7 | ▪ 1 | - | - | ▪ Moderate: r = 0.467 | ▪ - |
| Kessel et al. | ACT: 200-225  aPTT: 80-110  anti-Xa: 0.3-0.7 | ▪ 1 | - | - | ▪ Strong: r = 0.62 (p < 0.001) | ▪ - |
| Liveris et al. | ACT: 180-220  aPTT: 55-75  anti-Xa: 0.3-0.7 | ▪ 2 | - | - | ▪ Moderate to strong positive correlation in 10 out of 17 patients. | ▪ - |
| McMichael et al. | ACT: -  aPTT: 60-80  anti-Xa: 0.4-0.7 | ▪ 4 | 0.42  (0.33-0.47) | 0.44  (0.38-0.48) | ▪ Weak: *ρ* = 0.1 (p < 0.001) | ▪ No: None of investigated anticoagulation monitoring tools (anti-Xa, aPTT and UFH dose) was associated with complications. |
| Meshulami et al. | ACT: -  aPTT: -  anti-Xa: - | ▪ - | - | - | ▪ - | ▪ Yes: Anti-Xa is associated with reduced haemorrhage (aOR 0.37, p = 0.02).  ▪ Anti-Xa is associated with better outcomes. |
| Moussa et al. | ACT: -  aPTT: -  anti-Xa: 0.2-0.4 | ▪ 3 | - | - | ▪ - | ▪ No: Daily minimum, maximum, and mean anti-Xa values are not associated with serious bleeding.  ▪ In comparison to aPTT, anti-Xa should be favorited. |
| Moynihan et al. | ACT: -  aPTT: -  anti-Xa: 0.4-0.6 | ▪ 4 | 0.32  (0.05-0.59) | 0.43  (0.17-0.68) | ▪ Weak: *r* = 0.364 (p <0.001)  - Absolute results from anti-Xa and TEG showed the best correlation with UFH dose. | ▪ No. |
| Nankervis et al. | ACT: 160-200  aPTT: -  anti-Xa: - | ▪ 1 | - | - | ▪ Strong: r = 0.75 (p < 0.001) | ▪ - |
| Nguyen et al. | ACT: 180-220  aPTT: 45-80  anti-Xa: 0.3-0.7 | ▪ 1 | - | - | ▪ Weak: r = 0.39 (p < 0.001) | ▪ - |
| Niebler et al. | ACT: 180-200  aPTT: -  anti-Xa: 0.5-0.7 | ▪ 2 | - | - | ▪ - | ▪ Yes: Time to first surgical exploration was longer in patients with anti-Xa than ACT  ▪ The incidence of intracranial haemorrhage was lower in anti-Xa group (p = 0.049)  ▪ Anti-Xa group had significantly lower blood loss and the blood products transfusion.  ▪ Anti-Xa protocol showed improvement in survival and a decrease in bleeding occurrence. |
| O’Meara et al. | ACT: -  aPTT: -  anti-Xa: 0.4-0.8 | ▪ 4 | - | - | ▪ Strong: *r* = 0.68 (p <0.01) | ▪ Anti-Xa protocol is possible, decreases sampling of blood, UFH infusion adaptations, and no increase in adverse events rate. |
| Perez Ortiz et al. | ACT: 160-180  aPTT: -  anti-Xa: - | ▪ 2 | 0.28  (0.09-0.75) | 0.27  (0.09-1.9) | ▪ Weak: *r* = 0.38 (p <0.001)  ▪ Anti-Xa assay correlates better with UFH compared to ACT. | ▪ No: No significant difference in anti-Xa levels (p = 0.42). |
| Rabinowitz et al. | ACT: -  aPTT: -  anti-Xa: - | ▪ 2 | - | - | ▪ Significant correlation of UFH doses and anti-Xa (R2 = 0.28, p < 0.001). | ▪ - |
| Sleeper et al. | ACT: -  aPTT: 80-100  anti-Xa: 0.35-0.7 | ▪ 1 | 0.3 | 0.3 | ▪ - | ▪ No: Bleeding was not associated with anti-Xa (OR = 0.91, 95% CI 0.75 – 1.10, p = 0.33). |
| Sulkowski et al. | ACT: 180-220  aPTT: -  anti-Xa: 0.3-0.8 | ▪ 2 | - | - | ▪ Moderate: r = 0.56 (p = 0.07) | ▪ - |
| Yabrodi et al. | ACT: -  aPTT: -  anti-Xa: 0.3-0.7 | ▪ 4 | - | - | ▪ Weak: r = 0.127 (p = 0.004)  ▪ Strong in patients with more than 8 sets of measurements: r = 0.651(p = 0.015). | ▪ - |

^a^Values presented as mean with standard deviation or median with interquartile range in IU/mL; ^b^Correlation: strong >0.6; moderate 0.4-0.6; weak <0.4; ^c^Information obtained through the corresponding author; UFH unfractionated heparin, *ACT* activated clotting time, *aPTT* activated partial thromboplastin time.

# Table S7. Reported adverse events associated with ECMO in the included studies (n = 26)

| **Author** | **Bleeding** | **Major bleeding (ELSO)** | **Intracerebral hemorrhage** | **Pericardial hemorrhage** | **Pulmonary hemorrhage** | **Surgical site bleeding** | **Cannulation site bleeding** | **Gastrointestinal bleeding** | **Thrombosis** | **CNS infarction** | **Deep vein thrombosis** | **Limb ischemia** | **ECMO circuit and membrane clot** | **Death on ECMO** | **Death during ICU stay** | **In-hospital mortality** |
| --- | --- | --- | --- | --- | --- | --- | --- | --- | --- | --- | --- | --- | --- | --- | --- | --- |
| Al-Jazairi et al. | 10 | 4 | 1 | - | - | - | - | - | 10 | 0 | 0 | - | 6 | - | - | - |
| Arnouk et al. | - | 9 | 1 | - | 1 | 3 | - | 3 | 5 | - | - | - | - | 7 | - | 18 |
| Bembea et al. | 15 | 15 | 7 | - | 7 | - | - | - | 20 | - | - | - | 20 | - | - | 11 |
| Delmas et al. | - | - | - | - | - | - | - | - | - | - | - | - | - | - | - | - |
| Descamps et al. | 35 | 23 | 9 | 3 | 2 | - | 14 | 1 | 28 | 10 | 11 | 3 | 6 | - | - | - |
| Deshpande et al. | 33 | - | 16 | - | 4 | - | - | - | 49 | - | - | - | 48 | - | - | 55 |
| Drop et al. | 20 | - | 9 | - | - | - | - | - | 17 | - | - | - | 13 | - | - | 36 |
| Feih et al. | 36 | 36 | - | - | - | - | - | - | 14 | 7 | 1 | - | - | - | 46 | 47 |
| Figueroa Villalba et al. | - | - | - | - | - | - | - | - | - | - | - | - | - | - | - | - |
| Henderson et al. | 16 | - | - | - | - | - | - | - | 27 | - | - | - | 10 | - | - | - |
| Hohlfelder et al. | 18 | - | - | - | - | - | - | - | 14 | - | - | - | - | - | - | 26 |
| Kessel et al. | 1 | - | - | - | - | 1 | - | - | 5 | - | - | - | 4 | - | - | - |
| Liveris et al. | - | - | - | - | - | - | - | - | - | - | - | - | - | 3 | - | 5 |
| McMichael et al. | 24 | - | 13 | - | - | - | - | 3 | 29 | 6 | - | - | 23 | - | - | 27 |
| Meshulami et al. | - | - | - | - | - | - | - | - | - | - | - | - | - | - | - | - |
| Moussa et al. | 150 | 150 | 6 | 79 | 9 | - | 46 | 10 | 87 | 46 | - | 15 | 26 | 103 | 126 | 136 |
| Moynihan et al. | 7 | - | - | - | - | - | - | - | 21 | - | - | - | 9 | - | 7 | - |
| Nankervis et al. | - | - | - | - | - | - | - | - | - | - | - | - | - | - | - | - |
| Nguyen et al. | - | - | - | - | - | - | - | - | - | - | - | - | - | - | - | - |
| Niebler et al. | 50 | - | 50 | - | - | - | - | - | 18 | - | - | - | - | - | - | - |
| O’Meara et al. | 6 | - | 1 | - | - | - | - | - | 7 | - | - | - | 7 | - | - | 11 |
| Perez Ortiz et al. | 8 | - | 1 | - | 1 | - | 6 | - | 4 | - | - | - | 4 | 4 | - | 9 |
| Rabinowitz et al. | - | - | - | - | - | - | - | - | - | - | - | - | - | - | - | 13 |
| Sleeper et al. | 25 | - | - | - | - | - | - | - | - | - | - | - | - | - | - | 16 |
| Sulkowski et al. | - | - | - | - | - | - | - | - | - | - | - | - | - | 4 | - | 8 |
| Yabrodi et al. | - | - | - | - | - | 25 | - | 4 | 20 | - | - | - | - | - | - | 65 |

*ECMO* extracorporeal membrane oxygenation, *ELSO* Extracorporeal Life Support Organization, *ICU* intensive care unit

# Figure S1. Funnel plot of publication bias


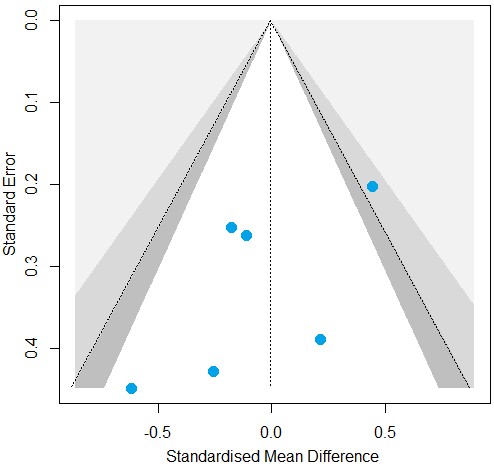


# Figure S1.1. Influence analysis: Baujat plot showing each study contribution to the overall heterogeneity


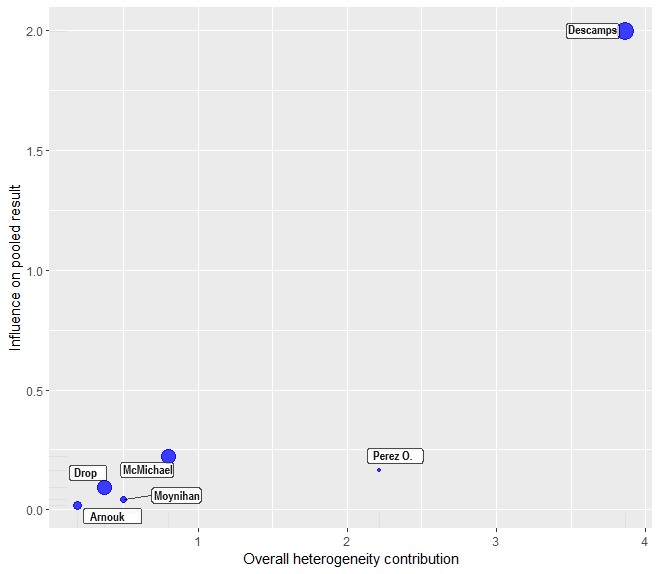


# Figure S1.2. Influence analysis: Studies sorted by the effect size


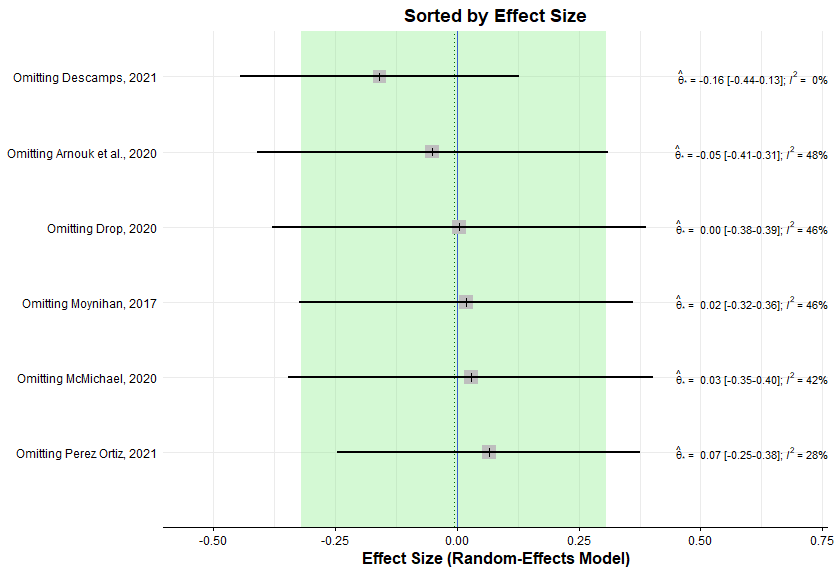


# Figure S1.3. Influence analysis: Studies sorted by the *I^2^*


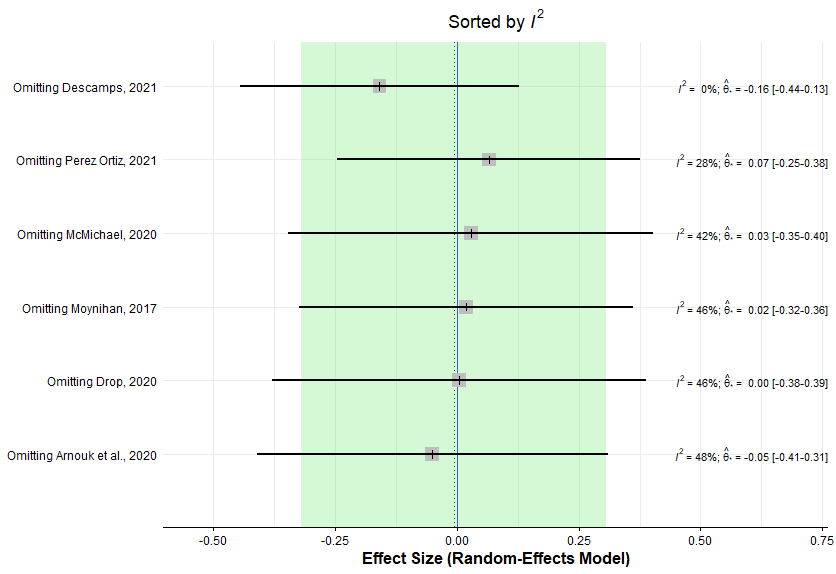


# Figure S1.4. Influence analysis


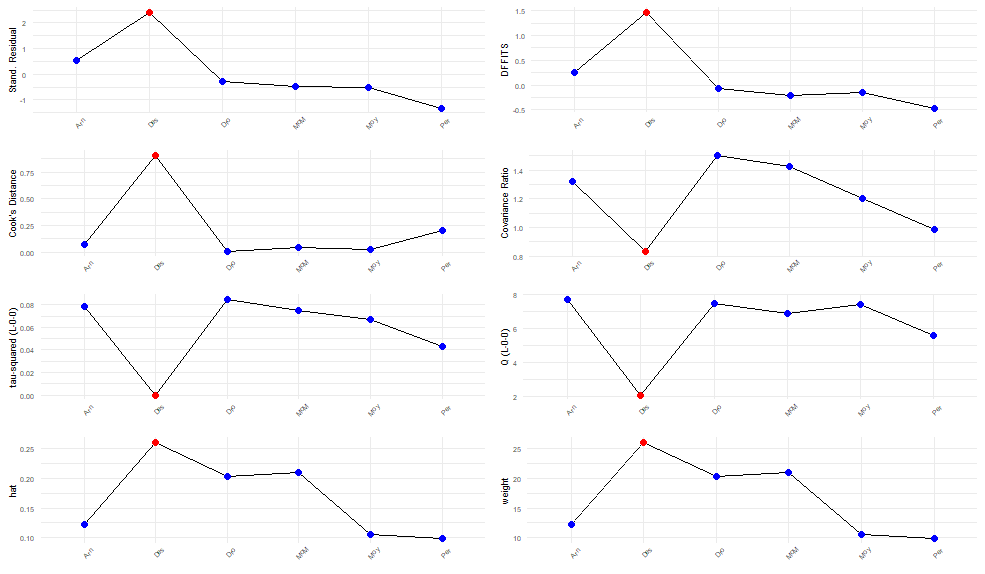


# Figure S2.1. Forest plot: Average anti-Xa values among adult patients with and without haemorrhagic event. ECMO extracorporeal membrane oxygenation.


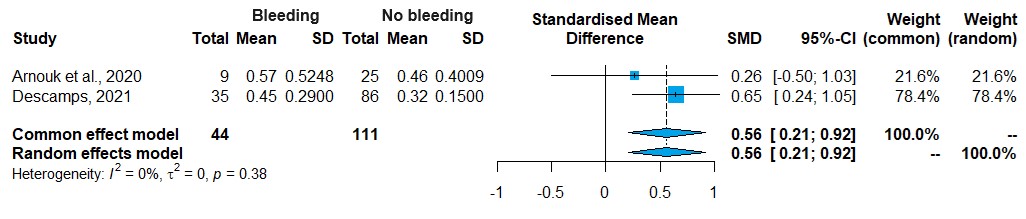


# Figure S2.2. Funnel plot of publication bias (studies including adult population).


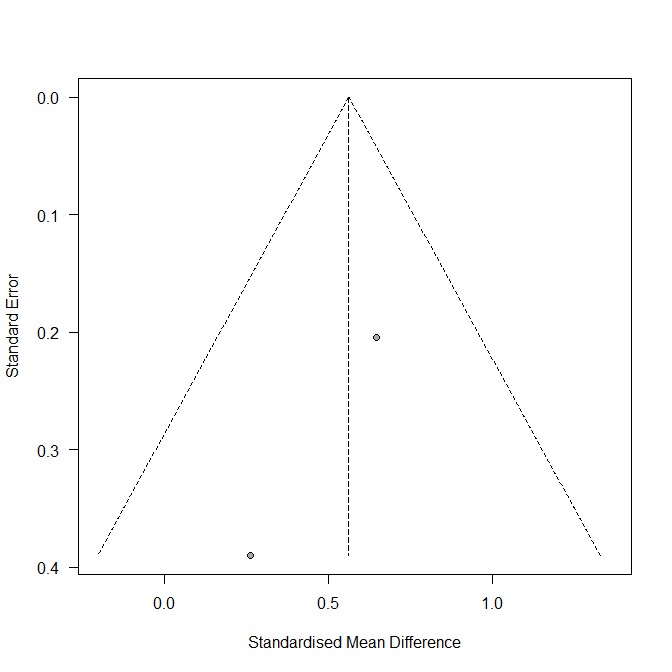


# Figure S2.3. Forest plot: Meta-analysis of correlation coefficients (studies including adult population).


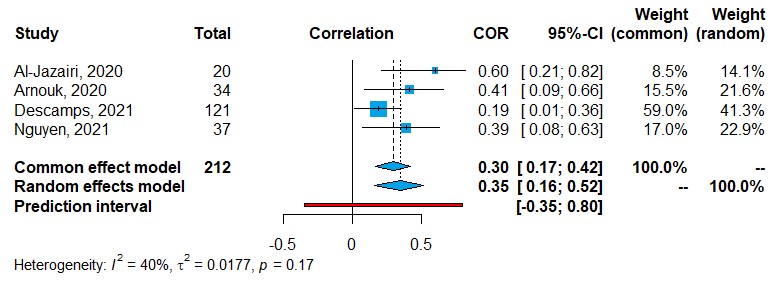


# Figure S3.1. Forest plot: Average anti-Xa values among paediatric patients with and without haemorrhagic event.


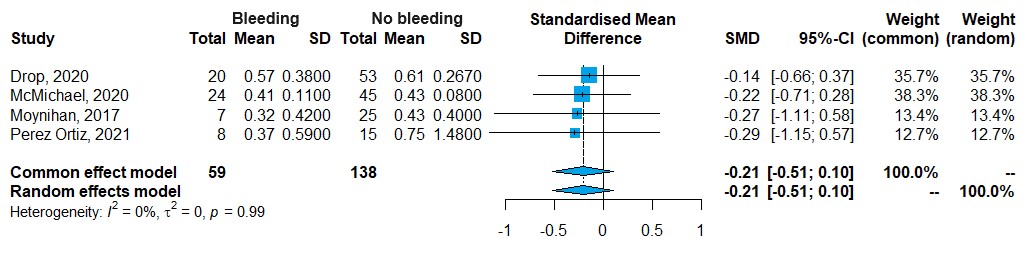


# Figure S3.2. Funnel plot of publication bias (studies including pediatric population).


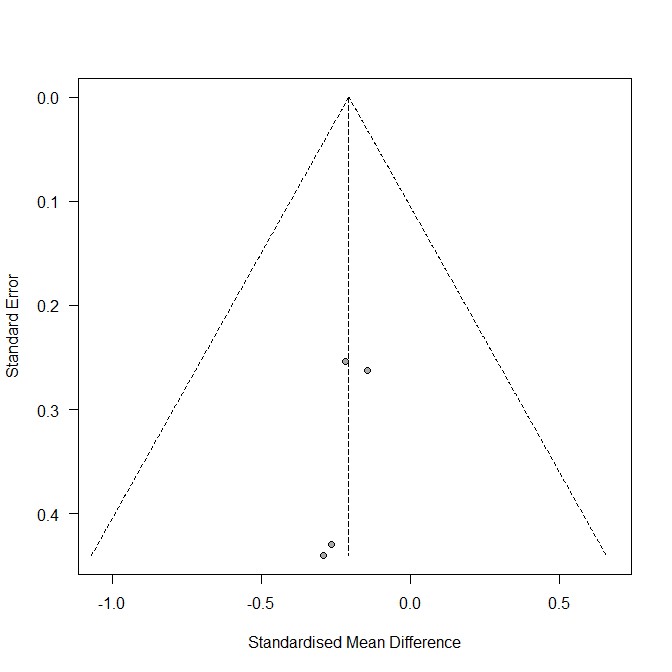


# Figure S3.3. Forest plot: Meta-analysis of correlation coefficients (studies including paediatric population).


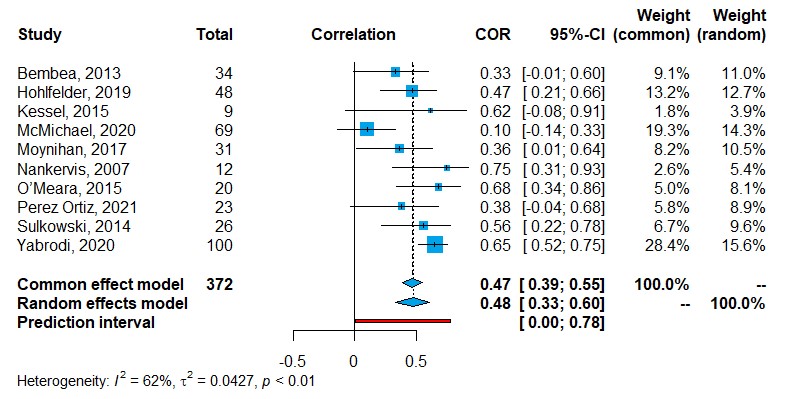


# Figure S4. Meta-analysis of correlation coefficients: Funnel and Baujat plot of publication bias


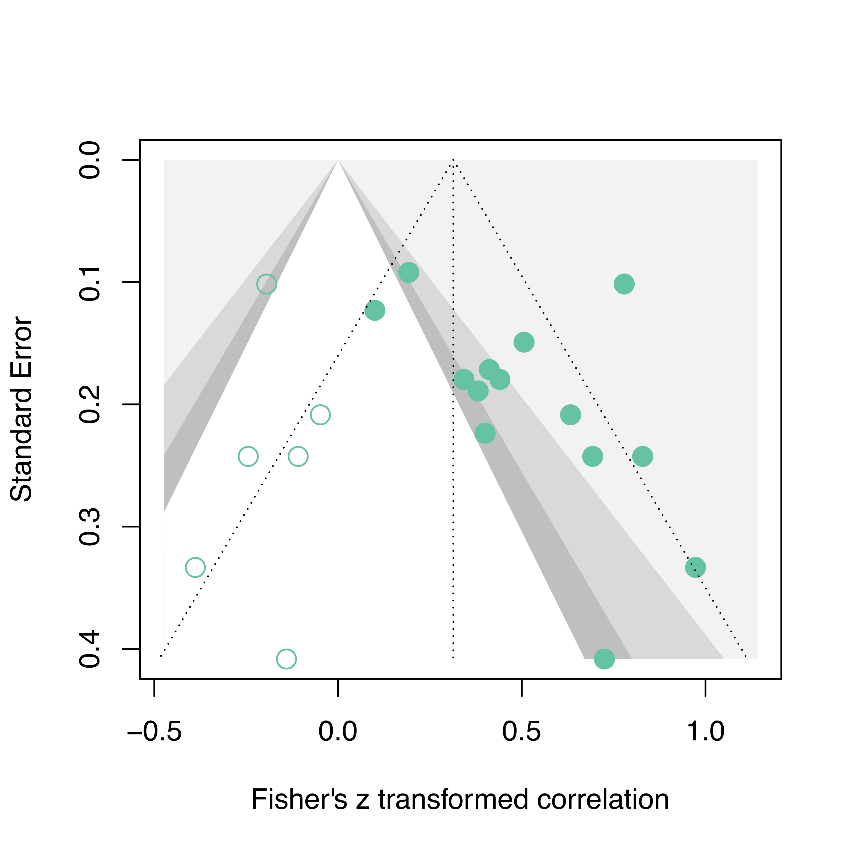

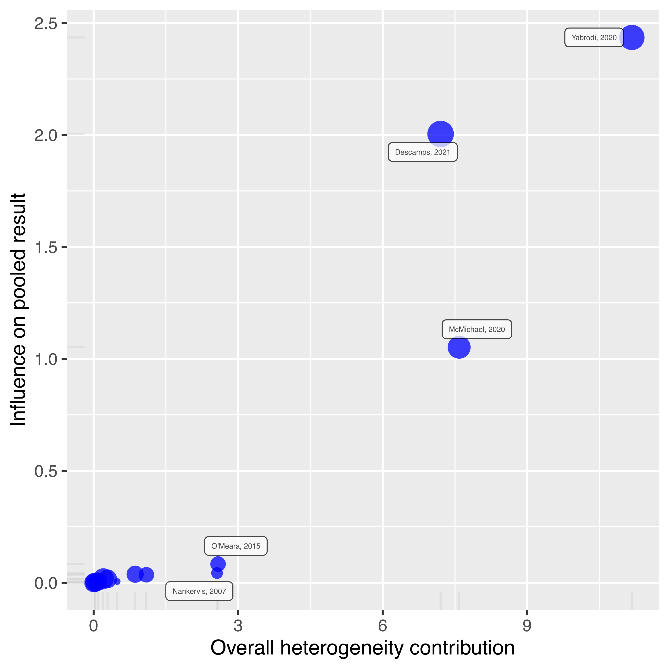


# Figure S5. Meta-analysis of correlation coefficients: Influence analysis


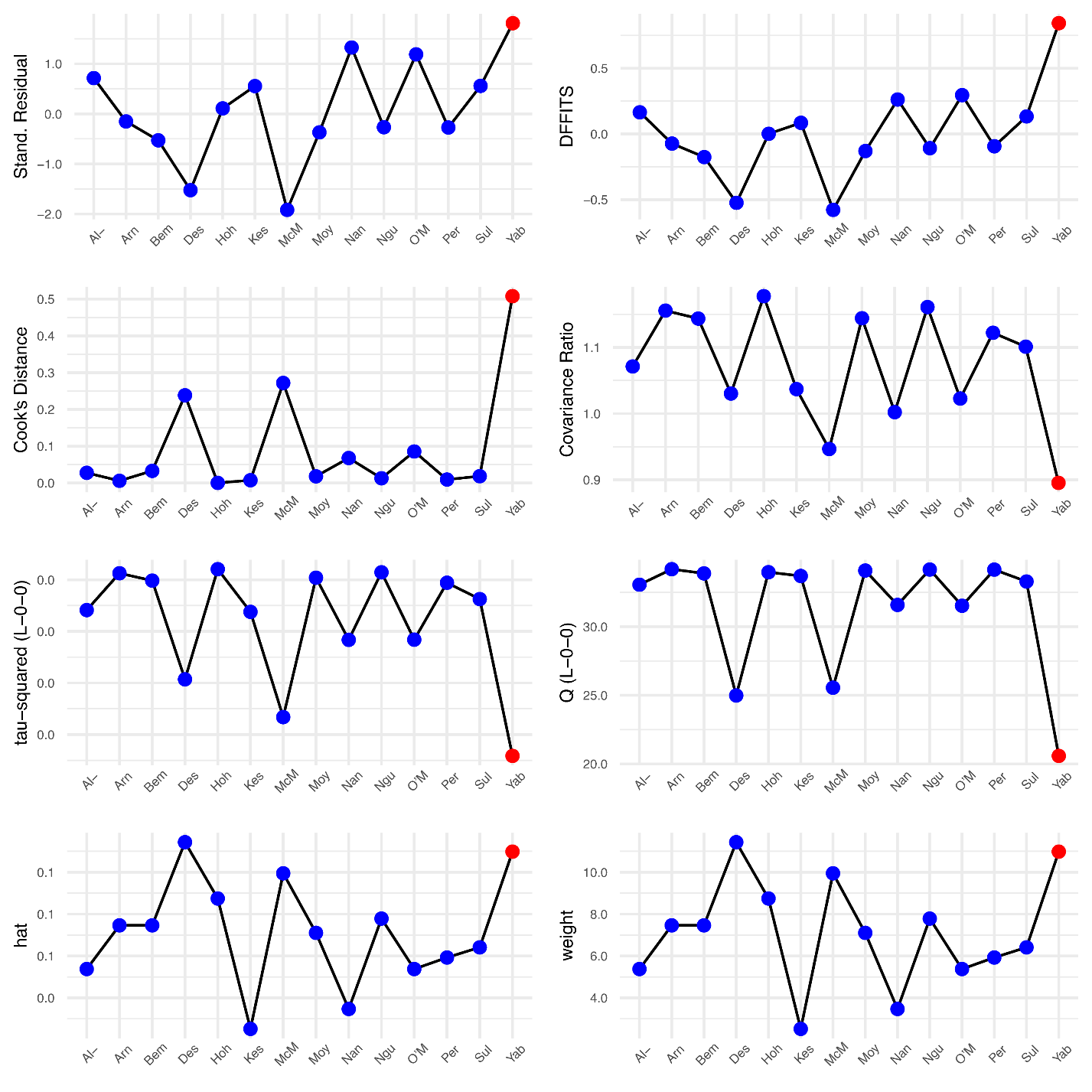


# Table S8. Meta-analysis of correlation coefficients: Influence analysis of included studies

| **Analysis** | **Proportion (95% CI)** | **95% Prediction Interval** | ***I^2^* (95% CI)** |
| --- | --- | --- | --- |
| Main Analysis | 0.44 (0.33; 0.55) | 0.03; 0.73 | 62% (32; 78) |
| Influential case removed  (Yabrodi et al., 2020) | 0.40 (0.29; 0.51) | 0.06; 0.66 | 42% (0; 70) |
